# Supplementary material for: Giant electrostriction-like response from defective non-ferroelectric epitaxial BaTiO3 integrated on Si (100)
Source: Nat Commun. 2024 Feb 16;15:1428. doi: 10.1038/s41467-024-45903-x (PMC10873356; doi:10.1038/s41467-024-45903-x)
Supplement: Supplementary file 1 — Supplementary Information [file 41467_2024_45903_MOESM1_ESM.pdf]

Supplementary Information

Giant electrostriction-like response from defective non-ferroelectric epitaxial $BaTiO_3$ integrated on Si (100)

Shubham Kumar Parate[1]*, #, Sandeep Vura[1]*, #, Subhajit Pal[1,2], Upanya Khandelwal[1], Rama Satya Sandilya Ventrapragada [1], Rajeev Kumar Rai[1,3], Sri Harsha Molleti[1], Vishnu Kumar[1], Girish Patil [1], Mudit Jain[1], Ambresh Mallya[1], Majid Ahmadi[4], Bart Kooi[4,5], Sushobhan Avasthi[1], Rajeev Ranjan[6], Srinivasan Raghavan[1], Saurabh Chandorkar[1], Pavan Nukala[1]#

Author Affiliations

[1]. Center for Nano Science and Engineering, Indian Institute of Science, Bengaluru-560012, India

[2]. School of Engineering and Materials Science, Queen Mary University of London, London E1 4NS, United Kingdom

[3]. Materials Science and Engineering, University of Pennsylvania, 3231 Walnut Street, Philadelphia, 19104

[4]. Zernike Institute for Advanced Materials, University of Groningen, Groningen, 9747AG, The Netherlands

[5]. CogniGron center, University of Groningen, Groningen, 9747 AG, The Netherlands.

[6]. Materials Engineering, Indian Institute of Science, Bengaluru, 560012- India

*Represents equal contribution

#Corresponding Author Address

Shubham Kumar Parate, Center for Nano Science and Engineering, Indian Institute of Science, Bangalore-560012, India, email: shubhamkp@iisc.ac.in

Sandeep Vura, Center for Nano Science and Engineering, Indian Institute of Science, Bangalore-560012, India, email: sandeepv@iisc.ac.in

Pavan Nukala, Center for Nano Science and Engineering, Indian Institute of Science, Bangalore-560012, India, email: pnukala@iisc.ac.in

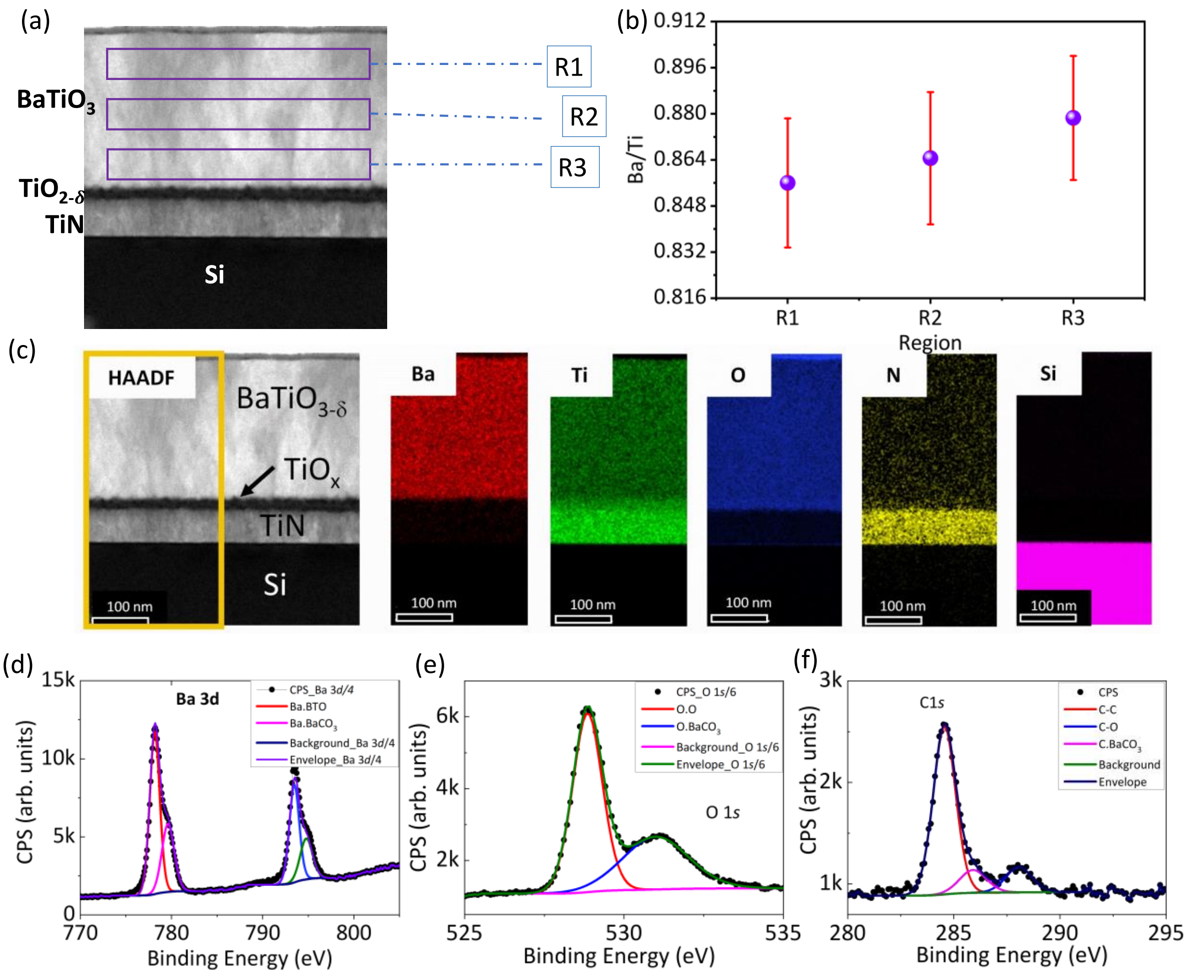

Supplementary Fig. 1: Chemical maps of various layers in the heterostructure: (a) cross section HAADF STEM of the BTO/TiN/Si stack after annealing in O_2 ambient showing different areas R1, R2 and R3, corresponding EDS atom% Ba/Ti ratio from the regions marked in (a) is shown in (b), (c) shows the EDS map corresponding to the HAADF STEM image showing elemental distribution (intensities correspond to atom%) in BTO, TiN, layers, and Silicon substrate. High Resolution XPS spectra of the unetched BTO surface post annealing with fits of (d) Ba 3*d* (e) O 1*s* (f) C 1*s*.

Supplementary Note 1: Supplementary Fig 1c shows the EDS map acquired on the cross-section FIB lamella of sample stack Barium Titanate (BTO) grown on Titanium Nitride (TiN) buffered Si (100). It can be seen that an additional TiO_x interfacial layer of thickness ~20 nm is formed between TiN and BTO.

STEM EDS spectrum quantification of Ba/Ti ratio (Brown-Powell method) at various depths into the BTO film from the surface is shown in Supplementary Fig 1a and b. It can be seen from Supplementary Fig 1a and b, Ba/Ti ratio uniform throughout the film (if we ignore the first 2 nm at the surface). Ba/Ti~0.86 ±0.04 (precision error), which matches well with the values determined by XPS (Fig 1d-f). The cross-sectional maps of various elements including the interfacial layer are presented in Supplementary Fig 1c. Using both XPS and EDS we confirm that our films are homogenous in composition and significantly Ba deficient.

The XPS data from the surface (without etching) is shown in Supplementary Fig 1c and d). The Ba/Ti ratio on the surface (i.e., *w/o* etch) is 0.68 (±0.04), and it is indeed true that annealing and exposure to atmosphere changes the surface composition. We also observe the formation of $BaCO_3$ [1] on the surface which is evident from the high resolution XPS spectra of Ba 3*d*, O 1*s* and C 1*s* spectra as shown in Supplementary Fig 1.

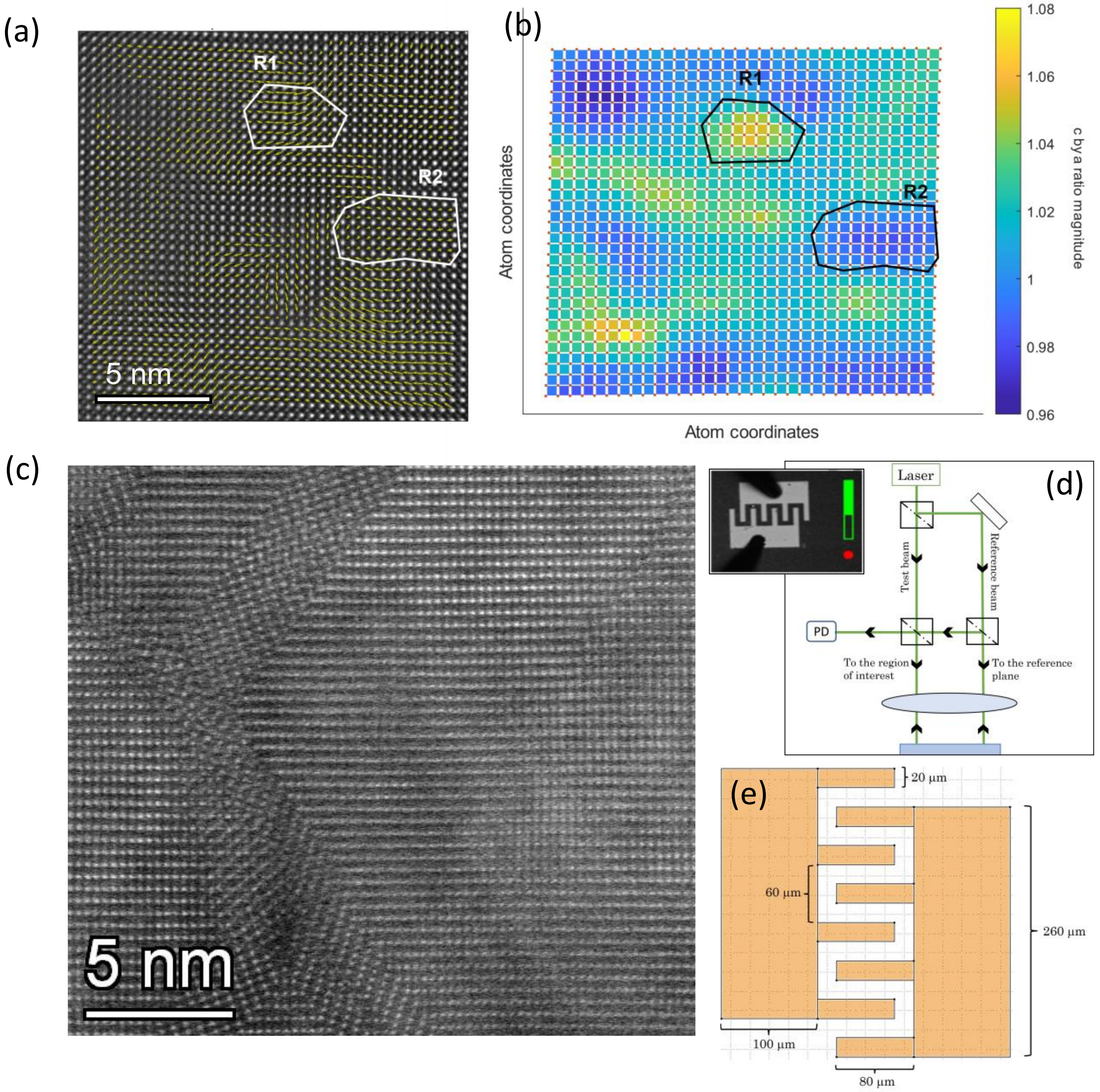

Supplementary Fig. 2: Lattice variation c/a ratio mapping, defects, electromechanical measurement scheme and device structure (a) Polarization map overlayed on HAADF STEM image also showing NPRs in white boxes (b) *c/a* ratio mapping on the same image shown in (a). Example NPRs are marked (c) HAADF-STEM image along [110] zone axis containing domain boundaries which could be one of the contributors to MW type of relaxation mechanisms coming from the BTO layer. (d) Laser doppler vibrometer setup and optical image (inset) of Interdigitated Electrodes and (e) corresponding detailed feature dimensions of IDE.

Supplementary Note 2: Here we present the polarization mapping and c/a ratio mapping on one of the high-resolution STEM images of BTO in Supplementary Fig 2a and b respectively. The arrows in Supplementary Fig 2a represents both the direction and the magnitude of Ti displacement and the colorbar in Supplementary Fig 2b corresponds to the magnitude of *c/a* ratio varying from 0.96 to 1.08. Although c/a maps also indicate the existence of nanoregions and correlate spatially with the NPRs shown by polarization mapping, one-to-one correlation between Ti displacements (defining NPR) and *c/a* ratio is not very clear. For e.g. we do find NPRs where *c/a*>1, yet the polarization is in-plane (R1, not tetragonal symmetry), and some where *c/a*<1 with in-plane polarization (R3)

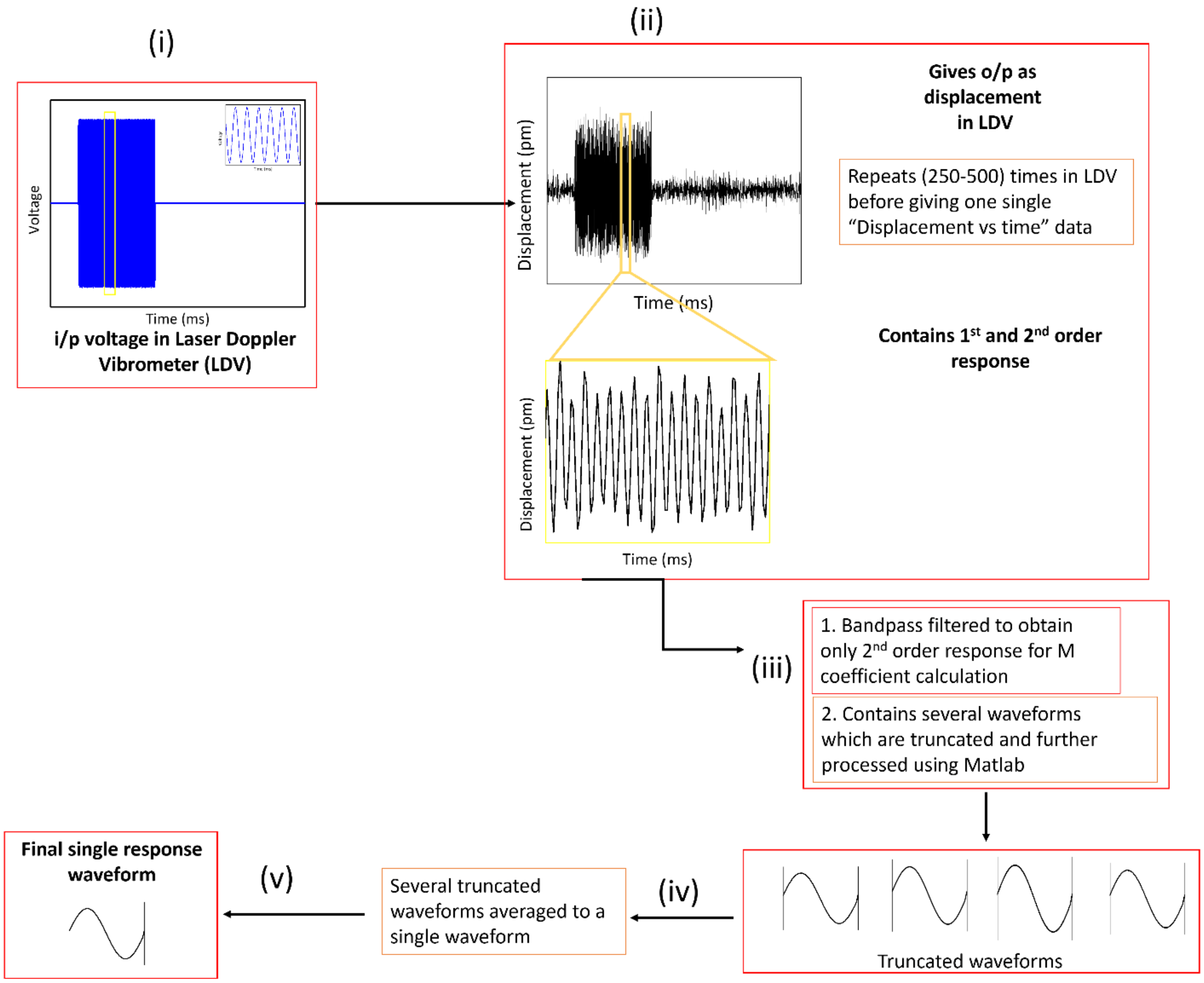

Supplementary Fig. 3 Data acquisition process and analyses: Data acquisition and analyses explained step-by-step with an example of an input AC waveform at 9 kHz.

Supplementary Note 3: The process for LDV data analyses is adopted as:

(i) We apply input waveform in the form of burst chirp for 30% (9-12 ms) of the total time duration (30-40 ms) as shown in Supplementary Fig 3 (i) in the schematic above.

(ii) We measure the corresponding displacement waveform as shown in (ii). This displacement is averaged over 250-500 bursts.

(iii) This burst-averaged displacement response is filtered to extract first and second harmonic responses separately.

(iv) The burst-averaged displacement contains displacement data acquired over many voltage cycles (for e.g., at 9 kHz, and ~9 ms acquisition, voltage vs time contains ~81 voltage cycles). The first harmonic and the second harmonic displacement values are further averaged across all these cycles at discrete number of voltage points, to obtain displacement vs voltage or strain vs field plots.

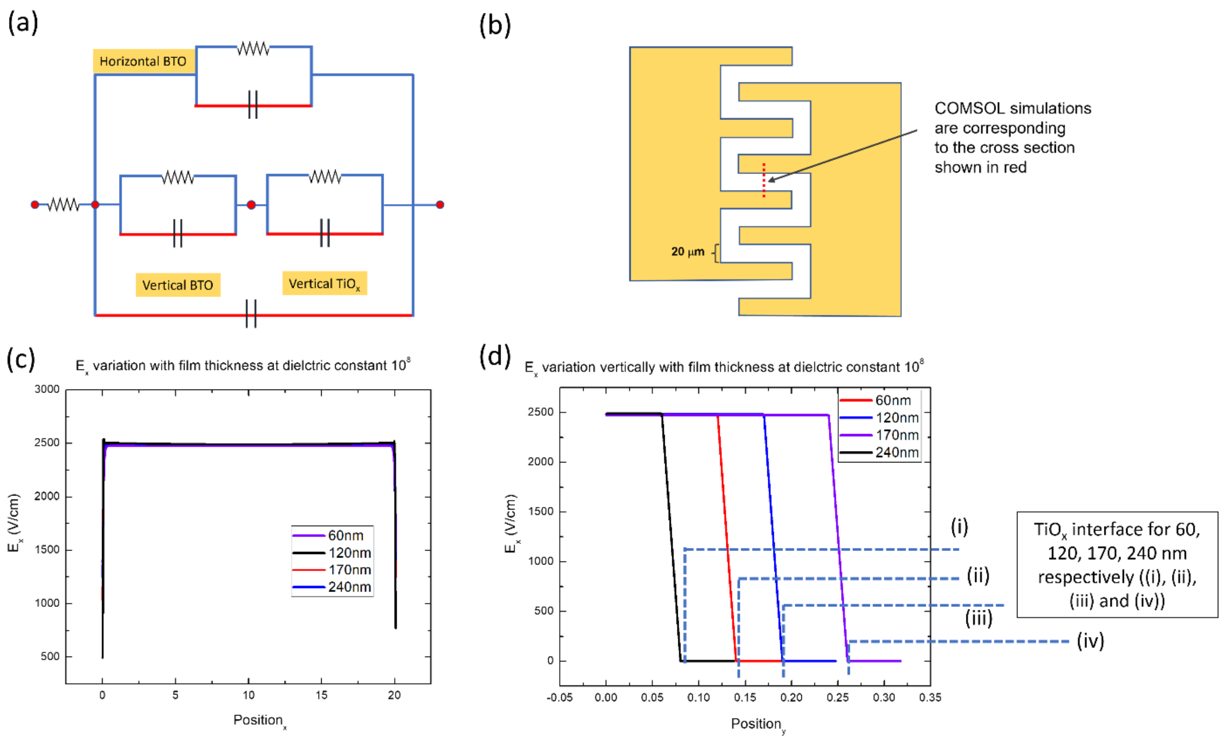

Supplementary Fig. 4: COMSOL simulation to study lateral field variation: (a) Equivalent circuit used for impedance fit on IDE device geometry (also refer Fig 4c and Supplementary Fig 11e and 12a), (b) The IDE schematic showing where the cross section (in red dotted line) has been taken for COMSOL simulations (c) lateral field (E_1 or E_x) variation across two lateral electrode and how increase in dielectric constant (as effective $\varepsilon_{r\text{-}apparent}$ is high) makes the field distribution uniform (d) distribution of field lines E_x or E_1 variation vertically at different film thickness.

Supplementary Note 4: Electric field in the horizontal direction (E_x or E_1) is simply calculated as

$$E_1 = \frac{Voltage\ (V)}{Separation\ of\ IDE\ electrodes\ (d)} \tag{1}$$

Here, we justify our assumption that E_1 (in plane component) is uniform spatially from source to the ground in the BTO layer. For this, we first fit our impedance spectroscopy data, performed on lateral IDEs by modelling the system using equivalent circuit as shown in Supplementary Fig 4a (also shown in Fig 4c, Supplementary Fig 11e and 12a). Using the extracted impedance values of different layers as the input, we performed COMSOL simulations to evaluate the in-plane field

(E_1) profile spatially in the BTO layer across the two electrodes, along a certain 2D cross-sections of the IDE (mentioned in Supplementary Fig 4b). Here we report results on this geometry (Supplementary Fig 4c), with BTO layer thickness of 60 nm, 120 nm, 170 nm and 240 nm, TiO_x layer thickness of 15 nm, and interelectrode lateral spacing of 20 μm. We take the ε_r of TiO_x as 15, which is consistent both with literature as well as our own impedance spectra of vertical devices. BTO layers ε_r is about 100 times larger (see impedance fits in Fig 4c and Fig S9e), however, leakage (or a parallel resistive path) makes it a layer with much lower apparent impedance. This renders the effective $\varepsilon_{r\text{-apparent}}$ of BTO layer (estimated from $|Z| = 1/\omega C_{apparent}$ as 1.79×10^8) to be 3×10^4 times greater than that of non-leaky BTO. At this large apparent dielectric constant of the BTO film, the field (E_1) becomes uniform across the device ($V_{applied}$=5 V), for all BTO thicknesses, making our assumption of estimating E_1 as 5 V/20 μm (2.5 kV/cm) very reasonable. E_1 also does not vary much vertically (out-of-plane) except near the TiO_x interface (Supplementary Fig 4d).

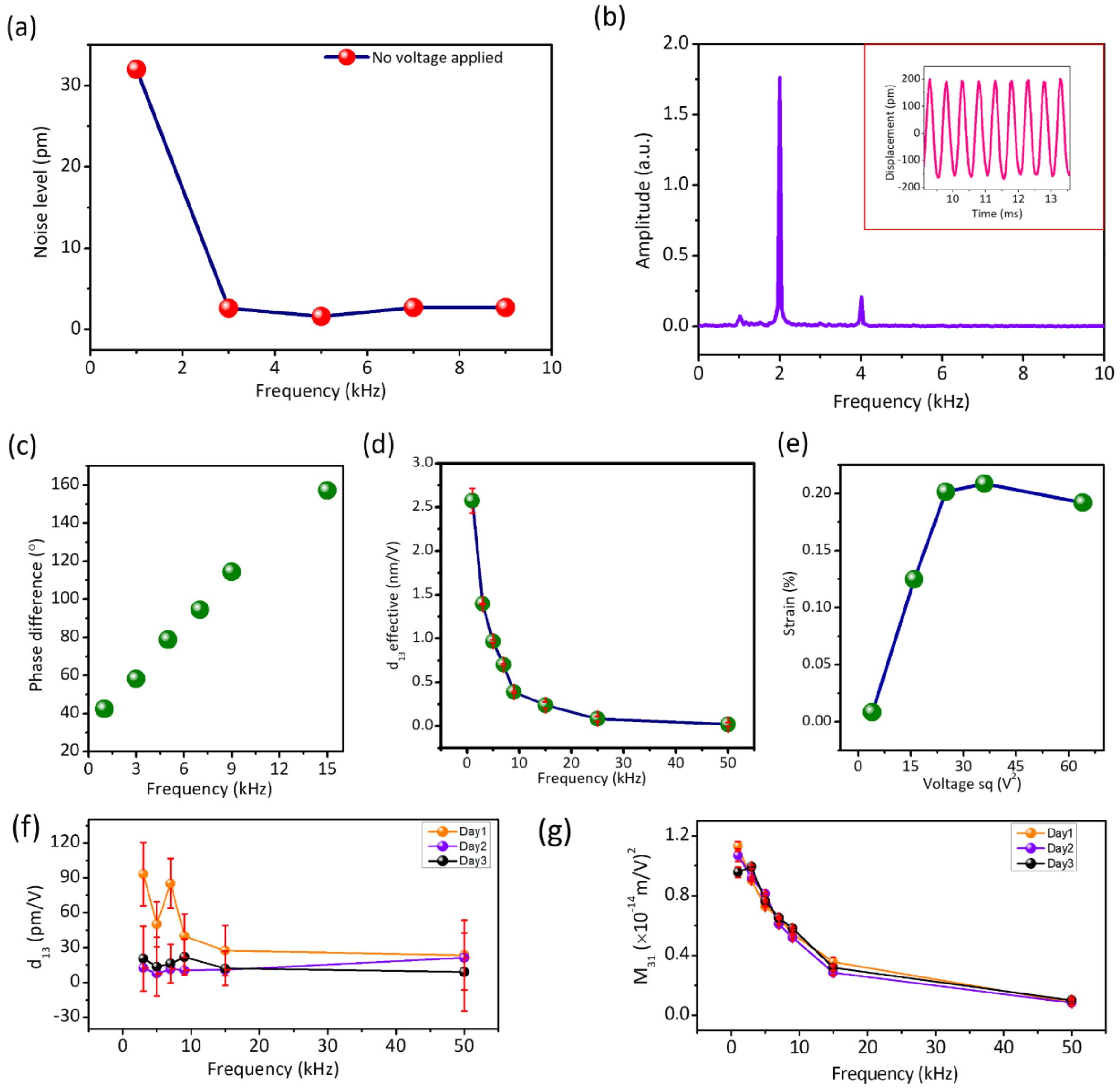

Supplementary Fig. 5: Measurement noise, higher harmonics, effective d, maximum strain saturation and repeatability study: (a) Noise level as a function of frequency without applying any voltage (b) Fourier analysis of the displacement signal (inset) obtained for an input voltage signal of 5V, 1kHz, showing strong 2nd and 4th harmonics, and weak first order and no 3rd harmonics (c) phase difference between square of voltage and second order displacement as a function of frequency (d) effective d_{13} (d_{13}^*) as a function of frequency, value at 1kHz is 2.57nm/V which is larger than d_{13} of lead based materials (e) maximum strain as a function of square of the applied voltage amplitude (f) The magnitude of first order piezoelectric component, d_{13} measured as a function of frequency on three different days keeps changing while in (g) the electrostrictive

M_{31} response is very consistent and as a result we do not focus on the d_{13}s. Error bars in Fig (d, f and g) represent ± SD obtained from averaging several measurements

Supplementary Note 5: Referring to Supplementary Fig. 5a, noise starts increasing below 1 kHz, which is a feature of the LDV, and this is why we do not report any measurements below 1 kHz. Our measured displacements at 1 kHz are in the order of 100s of pm across various IDE devices (3 and 5 V), which is why we report them.

Supplementary Note 6: In all our LDV measurements we use bandpass filter to remove other harmonic response (and get only 2nd harmonic displacement). However, if the bandpass range is large we do see higher harmonics such as that in Supplementary Fig 5b. We note that there is weak 1st harmonic response, large 2nd harmonic, no 3rd harmonic but a non-zero fourth harmonic (stronger than 1st harmonic) which is a feature of large electrostriction.

Supplementary Note 7: The effective d_{13} (d_{13}^*) coefficients were estimated as max strain/ max field. We show the variation of d_{13}^* as a function of frequency at 5V for the device whose M_{31} data is shown in Fig 3b. d_{13}^* is 2.57 nm/V at 1kHz and at higher frequency such as 9 kHz, it diminishes to 390 pm/V. These values are larger or comparable with Pb-based materials that show larger electrostrain.[2]

Supplementary Note 8: The maximum induced strain as a function of square of the applied voltage amplitude is shown in Supplementary Fig 5e. Until 5V, we see that the induced strain goes as voltage square, and then saturates. So in most of the devices the application of voltage was limited to V_{max} = 5 V.

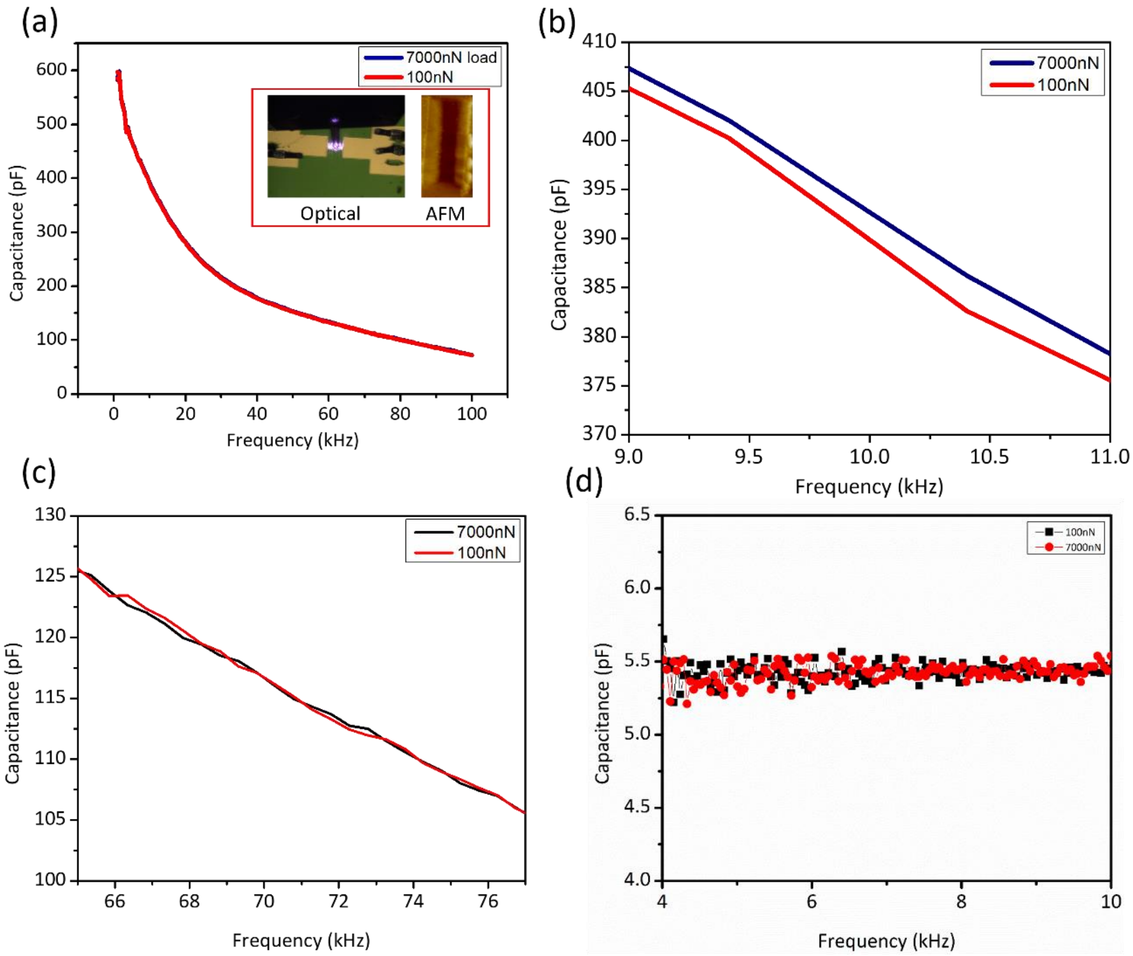

Supplementary Fig. 6: Another way of measuring M: (a) shows capacitance variation with frequency at two different loads (on IDE devices, inset), to view the subtle changes enlarged (b) view (low frequency) (c) view high frequency from plot (a), (d) nanoindentation on SiO_x using similar nanogap devices showing no change in capacitance with loading.

Supplementary Note 9: Preliminary indentation measurements to estimate M through the converse effect:

We performed static indentation at different loads on our samples using a 30 nm radius tip in an atomic force microscope. At every load, we measured capacitance vs frequency data on our device. However, these experiments were performed on lateral devices with a 800 nm nanogap between the source and ground (Supplementary Fig 6a inset, Optical image), and not our regular IDE geometry. The reasons to adopt a nanogap geometry are twofold:

(i) With a tip radius of 30 nm, we wanted to maximize the active area of the device that is loaded. This required minimizing the device lateral size to nanometric scale. In a limited time available, using e-beam lithography the smallest devices we were able to obtain had 800 nm gap size (Supplementary Fig 6a (inset, AFM image). We acknowledge that there is further room for device geometry improvement, which is why at the current moment we do not give any rigorous quantification of the material stress state, but rather approximate the stress to be applied load/tip area.

(ii) Having decided on a nanogap device geometry, the next question we answered is, what should be the electrode width (w) to gap length (l) ratio that would enable us to measure changes in capacitance upon application of load, given that our impedance analyser has sensitivity in pF. Here we show our simple calculations:

From thermodynamics, M can also be defined as follows:

$$M_{ijkl} = \frac{\partial(\chi_{ij}\varepsilon_0)}{\partial X_{kl}} \tag{2}$$

$$\frac{MA}{d} = \frac{\partial(\chi\,\varepsilon_0)}{\partial X}.\frac{A}{d} \tag{3}$$

$$\frac{MA}{d} = \frac{\partial(C)}{\partial X} \tag{4}$$

Where χ is the susceptibility, ε_0 is the absolute permittivity and X is the stress applied. Hence, as the referee correctly pointed out, the aim of this experiment is to measure changes in susceptibility (capacitance), with the application of stress (static).

$$\Delta C = \frac{MA\,\Delta X}{d} \tag{5}$$

We see from our preliminary indentation measurements that a load of 7 μN, which approximately corresponds to 2.4 GPa of stress, elastically deforms our BTO films, and the film regains its shape once the load is removed. We did not want to increase the load any further given the possibility of

plastic deformation or even tearing the film. Given the maximum ΔX = 2.4 GPa (Youngs modulus=80 GPa [3]), we constrain our A/d such that ΔC is measurable by our system (pico Farads). For M in the order of 10^{-15} m^2/V^2, A/d should be ~0.4 μm .

In a nanogap geometry with d = 800 nm (fixed by constraints mentioned in point (i)), based on lithography and lift off constraints, A was set to be 5 um x 200 nm.

However, in a lateral geometry, we can apply stress in the vertical direction through nanoindenter (X_{33}) and measure lateral capacitance (χ_{11}). From this we were able to estimate M_{1133} (M_{13} in Voigt notation). Applying in-plane stress to measure M_{31} is tricky. Although, we are designing experiments to do this, we will not be able to show this data currently (in such a short time). In the following, we will show that our starting experiments already show that M_{13} is also giant. We believe given the underlying lattice anharmonicity and dielectrically soft matrix (large tensor elements in susceptibility and mechanical compliance) it should not be surprising to expect that M_{13} and M_{31} are correlated.

We used AFM with cantilever tips of radius 30 nm for nano indenting BTO at static loads of 0.1 (35 MPa) and 7 μN (2.4 GPa), between the lateral electrodes. At every load we measured capacitance vs frequency (Supplementary Fig 6a) using an impedance analyser (MFIA-Zurich instruments).

For a load of 7 μN (stress applied is 2.4 GPa) the estimated ΔC = 3 pF, for M=10^{-15} m^2/V^2, and for 0.1 μN, ΔC is practically 0. Our *C vs f* plots between 9 and 11 kHz (Supplementary Fig 6b) show that ΔC measured is indeed 2-3 pF at 7 μN load. At higher frequency (65-80 kHz), we do not see any change in the capacitance upon loading (Supplementary Fig 6c), which also consistent with our interferometry (LDV) measurements of M coefficient at higher frequencies (less than the system sensitivity, see also Supplementary Fig 5a).

Also worth noting is the fact that we performed these experiments also on reference samples (SiO_x nanogap devices) and did not see any changes in capacitance with load of 7 μN (Supplementary Fig 6d). As we can see from Supplementary Fig 6d that the noise in capacitance measurement is about 1 pF, which puts a limit on the sensitivity of M from the indentation experiments to be ~2 x

10^{-16} m^2/V^2 with the current geometry. Any M less than this will not be measurable, as we see for data on our devices at higher frequencies (60-80 kHz), and also on the reference samples.
While these set of experiments already show another proof of BTO being a giant M electrostrictor, still only a part of the film sees the load of the indenter. There is further room for improvement before quantifying the M_{13} values that we obtain from these experiments, by a) using larger radius indenters with flat tips, and (b) further scaling down the device channel size by optimizing e-beam lithography.

Supplementary Note 10: On the vertical MIM stack device configuration, bending experiments can be performed to estimate M_{31}, by estimating the change in dielectric constant of the active layer (ε_{33}) with applied stress (X_{11}), through a converse effect. The important point here is that the dielectric constant (or capacitance) of the active layer ($BaTiO_3$) changes significantly with the application of stress. However, it must be noted that this active layer (static ε_r=1500 as per our impedance fits shown in Supplementary Fig 7a and b) is in series with a low dielectric constant (ε_r=15) TiO_x, a passive layer, which dominates the total capacitance of the stack. As we explain, this will lead to only small changes in the total capacitance, be it in an indentation experiment or a bending experiment.

To elucidate this, we show some back-of-the-notebook calculations of changes in stack capacitance to be expected (despite large changes in the capacitance of active layer) in a bending experiment with application of stress (100s of MPa) on a 120 nm thick film, in the following:

Given the device dimensions (A=100 × 100 μm^2, d_{BaTiO3}=120 nm, d_{TiOx}=20 nm), and material dielectric constants (static ε_{33}-BTO = 1500, static ε_{33}-TiO_x =15, see Supplementary Fig 7b), C_{TiOx}= 66.37 pF, C_{BTO} = 1100 pF. The stack capacitance will then be ($1/C=1/C_{BTO}+1/C_{TiOx}$) 62.59 pF, predominantly dominated by the low capacitance (high impedance) TiO_x layer. For this exercise, we ignore parallel resistors to these capacitors. If M_{31} (real part) of BTO is 10^{-15} m^2/V^2 (as reported), then at a stress of 650 MPa, change in dielectric constant of BTO is in the order of ~73000. Although, C_{BTO} (at 650 MPa) sees a huge change, the stack capacitance then is 65.92 pF, hardly a change by 3 pF from the unstressed conditions, again dominated by the low impedance TiO_x layer. The maximum stack capacitance is limited to 66.37 pF. Thus, we are looking at measuring changes in ~1-3 pF in the stack capacitance, irrespective of however huge the change

in BTO capacitance/dielectric constant is (as we observed earlier in the nanoindentation measurements). As we reported earlier in the m/s, the sensitivity of our capacitance measurement is ~1 pF. We performed the bending experiments, with a maximum stress of 650 MPa on the film. In this experiment, assuming that the only reason for capacitance change in BTO is due to its M_{31}, we estimate that the minimum M_{31} we can measure is ~10^{-16} m^2/V^2 (Supplementary Fig 7c). Indeed, our bending experiments show a change in stack capacitance by ~2 pF, which corresponds to real part of M_{31} of atleast 10^{-16} m^2/V^2 (also giant).

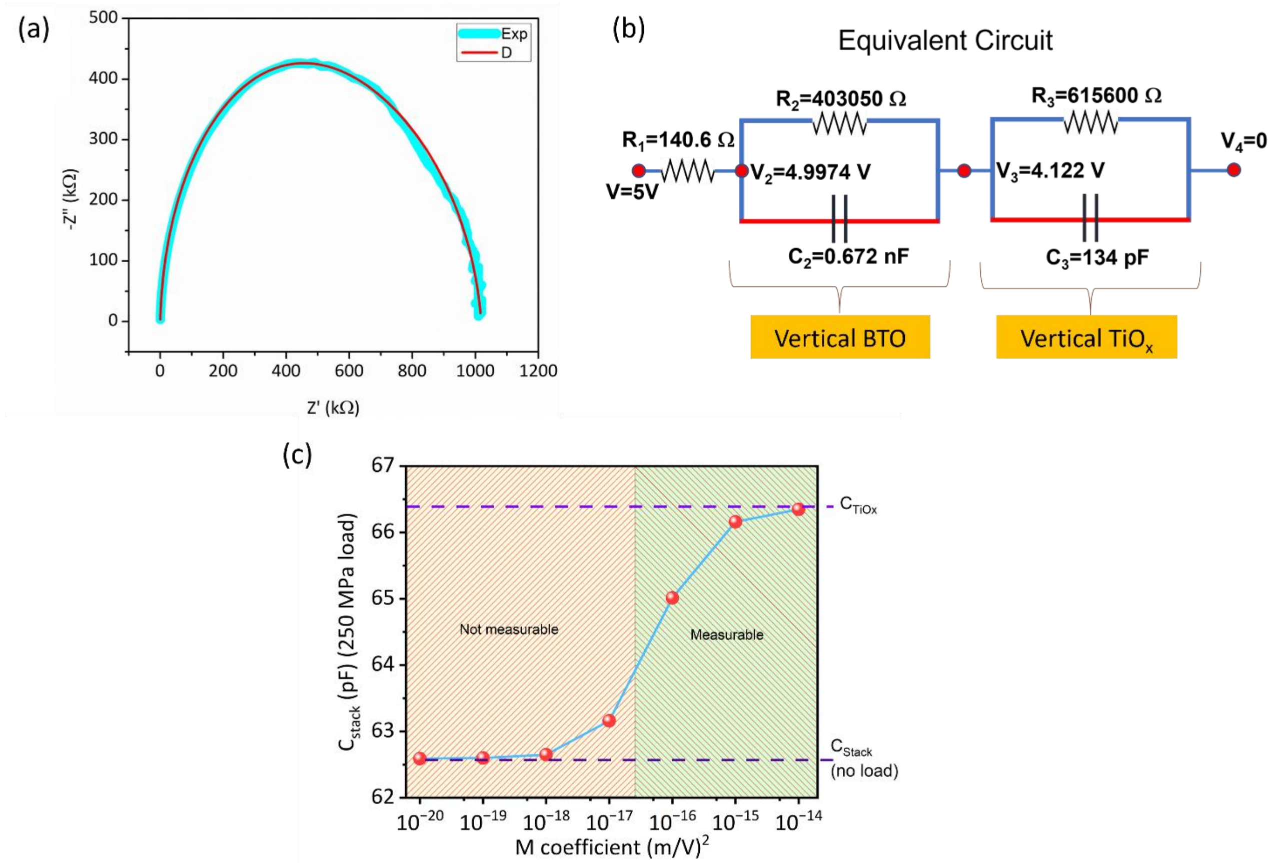

Supplementary Fig. 7: Impedance measurement on MIM devices: (a) Bode impedance plot for vertical MIM device and corresponding fit using equivalent circuit in (b), (b) shows the values of RC elements obtained after impedance fitting (c) shows how increase in *M* coefficients saturates the stack capacitance that can be practically measured.

Supplementary Fig. 8a shows the experimental set up with a fixture to bend the thin film stack on Si, while simultaneously performing impedance measurements. Initially, the sample (width = 5 mm) is exactly fit in the fixture. The fixture is subsequently tightened using a screw, which reduces

the distance between the two flat ends and thus bends the sample as shown in schematic in Supplementary Fig 8b. Motion of the screw by a pitch (360°) corresponds to reduction in the fixture dimension by 500 um. Thus, the curvature of the film is controlled by the angle by which screw is tightened. We perform our experiments by tightening the screw from 0° to 5°, which corresponds to maximum radius of curvature of 1/(32.5 m). Assuming elastic modulus of BTO = 80 GPa, this corresponds to a max stress of ~650 MPa.

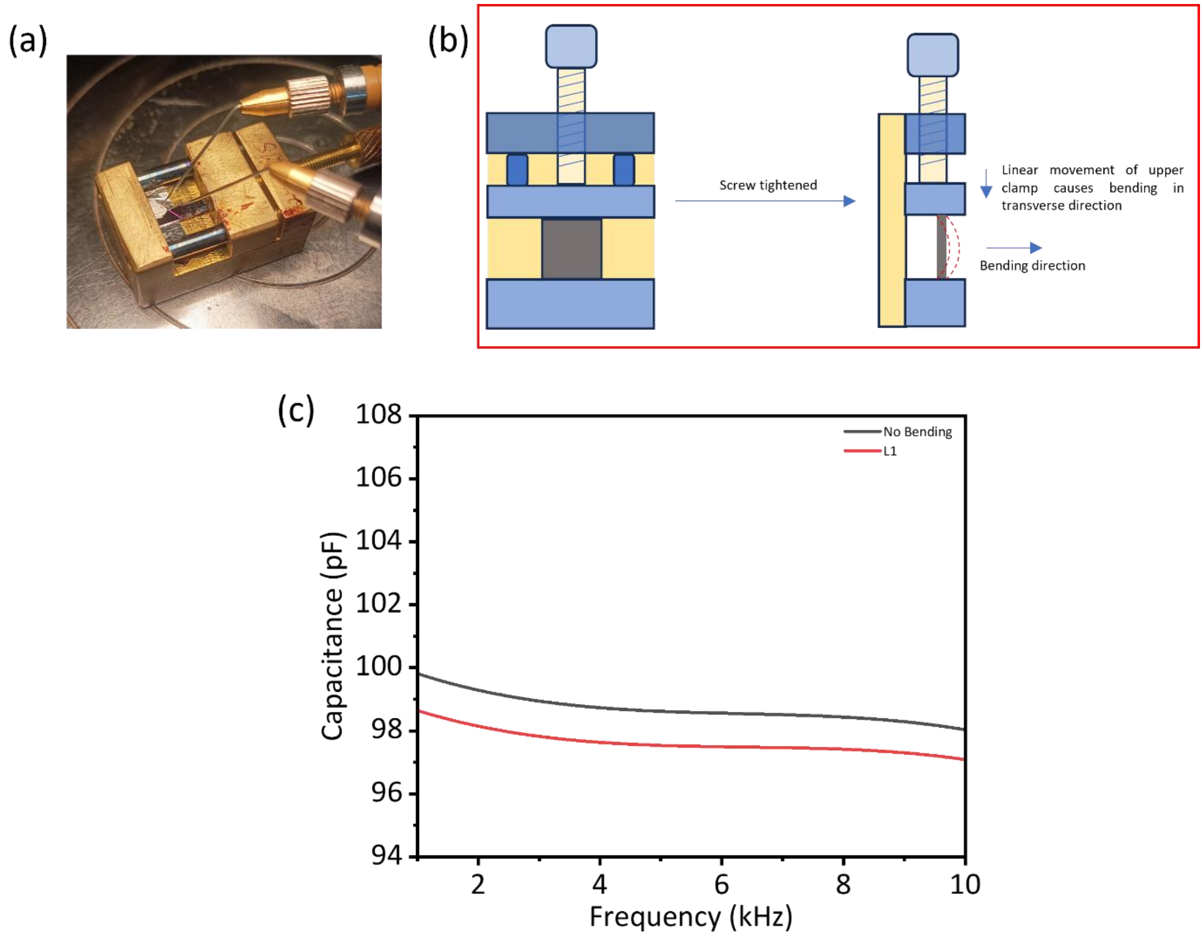

Supplementary Fig. 8: Bending experiment set-up and capacitance response: (a) Experimental setup showing a fixture set up used for bending our sample and simultaneously measuring impedance using two probes, (b) schematic showing how the load is applied using the setup in (a), (c) capacitance as a function of frequency under 'no loading'(no bending) and 'loading state'(L1).

In the frequency range of 1 to 10 kHz in Supplementary Fig 8c, we see that change in stack capacitance is ~1-2 pF. At 650 MPa of stress, this corresponds to M_{31} in the order 10^{-16} m^2/V^2.

This is also giant (much more than classical response of BTO), but an order of magnitude smaller than our direct electrostrain measurements. These bending experiments only give thermodynamic or real part of M_{31}. In the back-of-the notebook calculation, we assume ideal capacitors, no leakage, and unclamped conditions. The differences (~10^{-16} to 10^{-15} m^2/V^2) in the indirect and direct measurements can be because of all these factors.

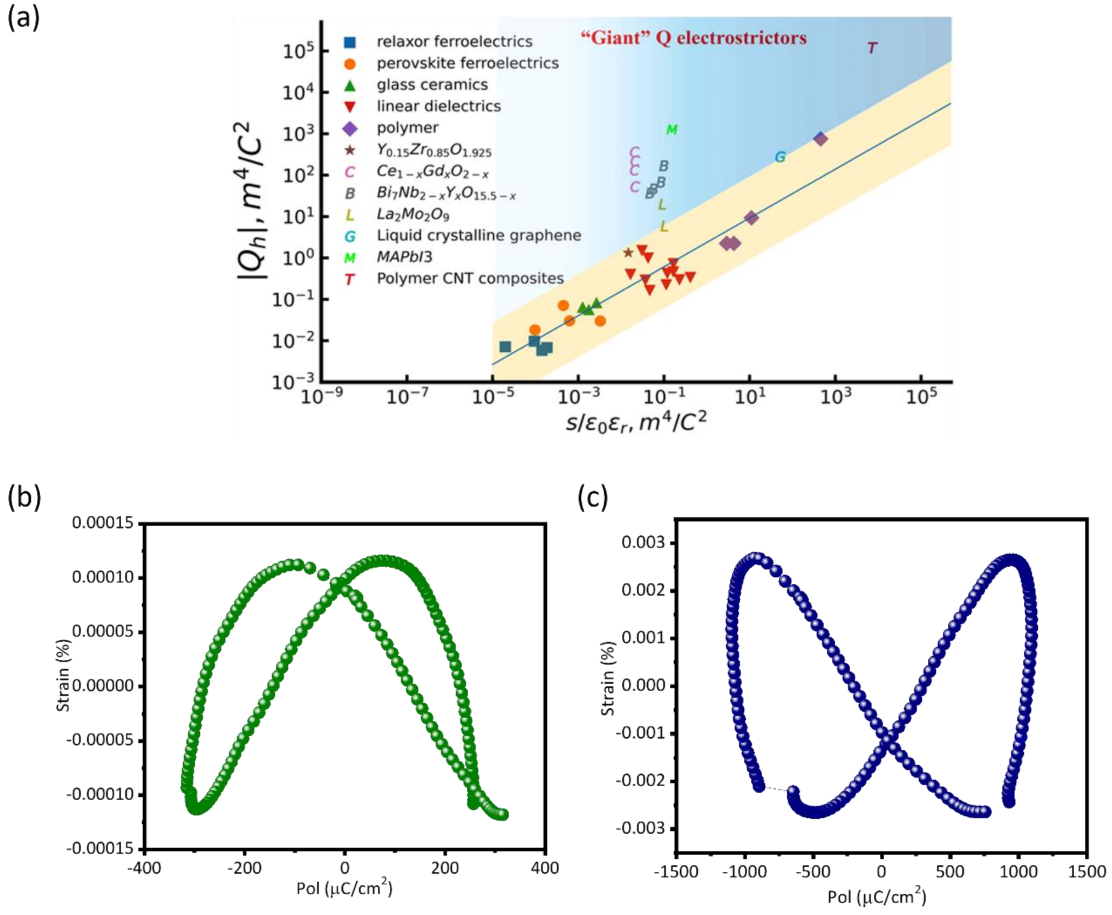

Supplementary Fig. 9: Q electrostriction response of defective BTO: (a) empirical consolidation of Q as a function of $s/\varepsilon_0\, \varepsilon_r$, where s is the compliance, and ε_r is the material dielectric constant, reproduced from reference [4]. Giant Q electrostrictors are the ones which show values of Q above the band. (b,c) Variation of second order strain (obtained from ε-t plots) with first order polarization (obtained from P-t), for (b) V_{max} = 1 V and (c) 3V at 5 kHz

Supplementary Note 11: We also estimated Q_{31} of our devices by measuring P-E loops and ε (strain)-E loops on the same device. Given that P is a non-linearly dependent on E, we use the following mathematical formulation to extract Q:

$$\varepsilon = QP^2 \quad (6)$$

If

$$E = E_0 \, sin(\omega t) \quad (7)$$

$$P = P_0 \, sin(\omega t) + P_1 \, sin(2\omega t), \quad (8)$$

given that the material does not have any spontaneous polarization. We address each of these terms as P_{ω}, $P_{2\omega}$, and so on. Second order strain $\varepsilon_{2\omega}$ is contained in QP_{ω}^{2}. Therefore, in phasor notation, we have:

$$\varepsilon_{33,0}(2\omega) = Q_{31} e^{-i\varphi} P_0^2 \quad (9)$$

In Supplementary Fig. 9b and c, we show the variation of second order strain (obtained from ε-t plots) with first order dielectric displacement (obtained from D-t), for V_{max}=1 V and 3V at 5 kHz. We restrict ourselves to V_{max}=3V, because beyond that leakage becomes significant even in less-leaky devices, and estimation of D_1 will be erroneous. However, it is still possible that values of D are overestimated, and so the Q_{31} values reported should be taken as the lower limits of the actual Q_{31} of the devices. We obtain $|Q_{31}|$ in the order of 10^{-7} $(m^2/C)^2$. By the definition of giant electrostrictors presented in reference [4] (see Supplementary Fig. 9a), our thin films are not giant Q electrostrictors. Giant "M" electrostrictors need not be giant "Q" electrostrictors.[4] This could be related to the easily polarizable and soft matrix, which enhances M but not Q.[4] Thus, in this manuscript, we do not further discuss values of Q_{31}, but only look at M_{31}.

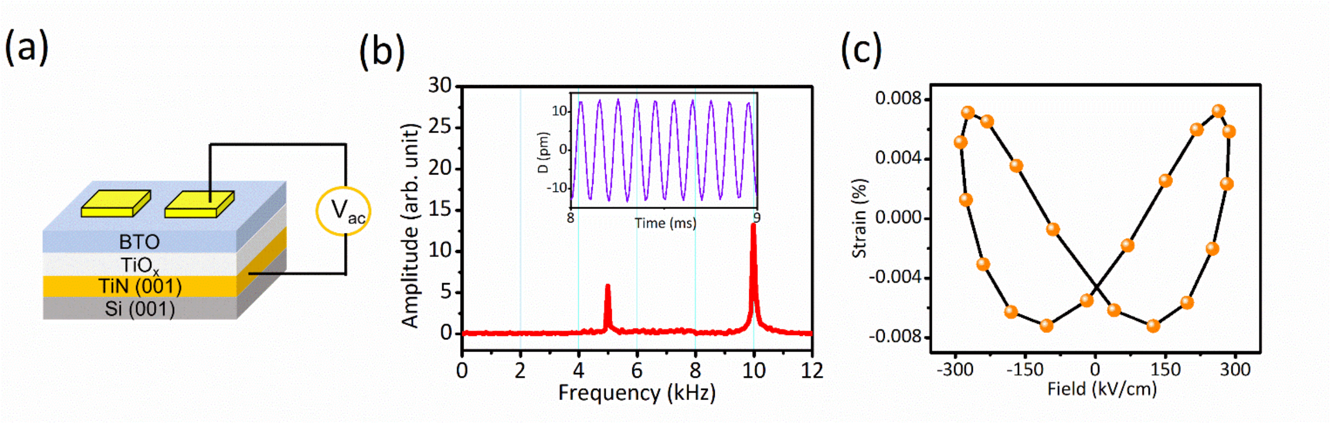

Supplementary Fig. 10: Electromechanical response on vertical capacitors. (a) Metal insulator metal (MIM) capacitor schematic. (b) Fast fourier transform and corresponding vertical 2nd order displacement-time response (inset) as a function of time of MIM capacitor with BTO thickness 170 nm. (c) Second harmonic response with a varying electric field (5 V) at 5 kHz.

Supplementary Note 12: Supplementary Fig 10a, b and c shows AC strain variation at 10 kHz (2nd order) for voltage input of 5V at 5 kHz. We note that the amplitude of oscillation is ~15 pm. This corresponds to M_{33} of 1.02×10^{-19} m^2/V^2. All the devices we measured had M_{33} in the same order of magnitude, which is close to its classical value. Thus, the giant electrostriction we report here is only in terms of M_{31} and for lateral devices, not for M_{33}.

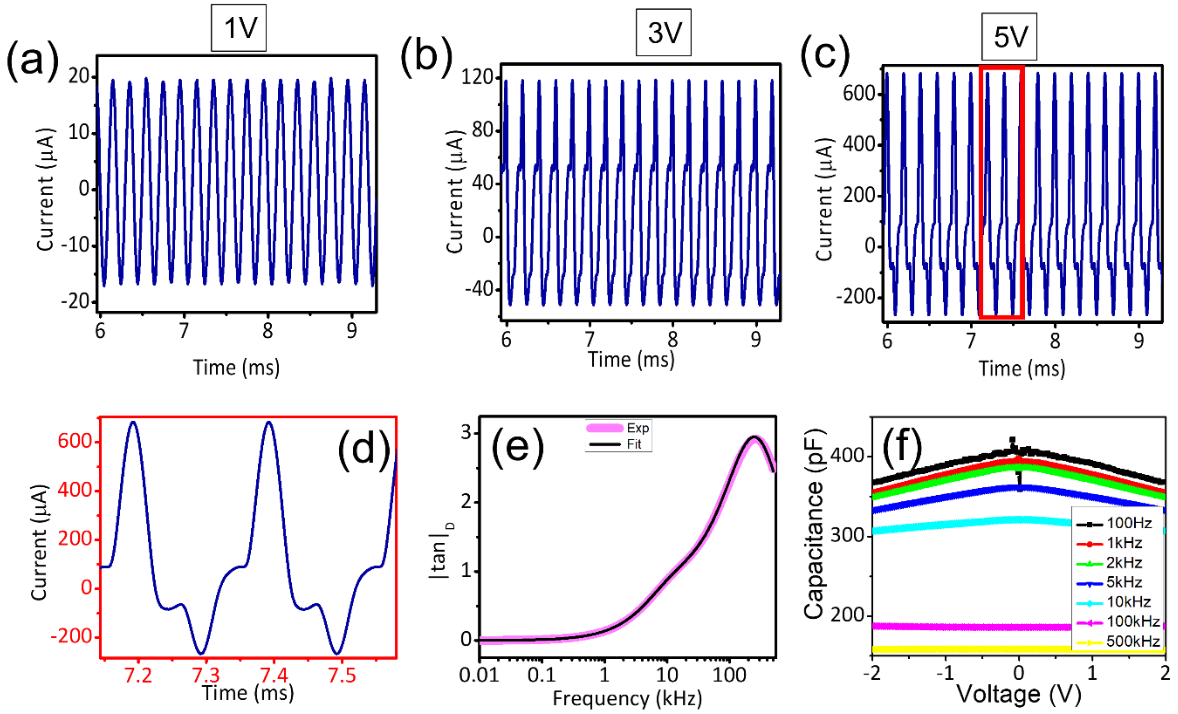

Supplementary Fig. 11: Large signal I-V and small signal C-V measurements. Current response as a function of time for three different voltages (a) at 1 V (b) 3 V, and (c) 5 V. (d) Magnified region from marked red enclosure in (c) showing non-linear current response. (e) Dielectric loss tangent tan δ and corresponding fit obtained for equivalent circuit. (f) *C-V* measurements as a function of frequency. Notably a peak at 0V in both forward and reverse sweeps upto 10 kHz indicates non ferroelectric, yet very soft dielectric response.

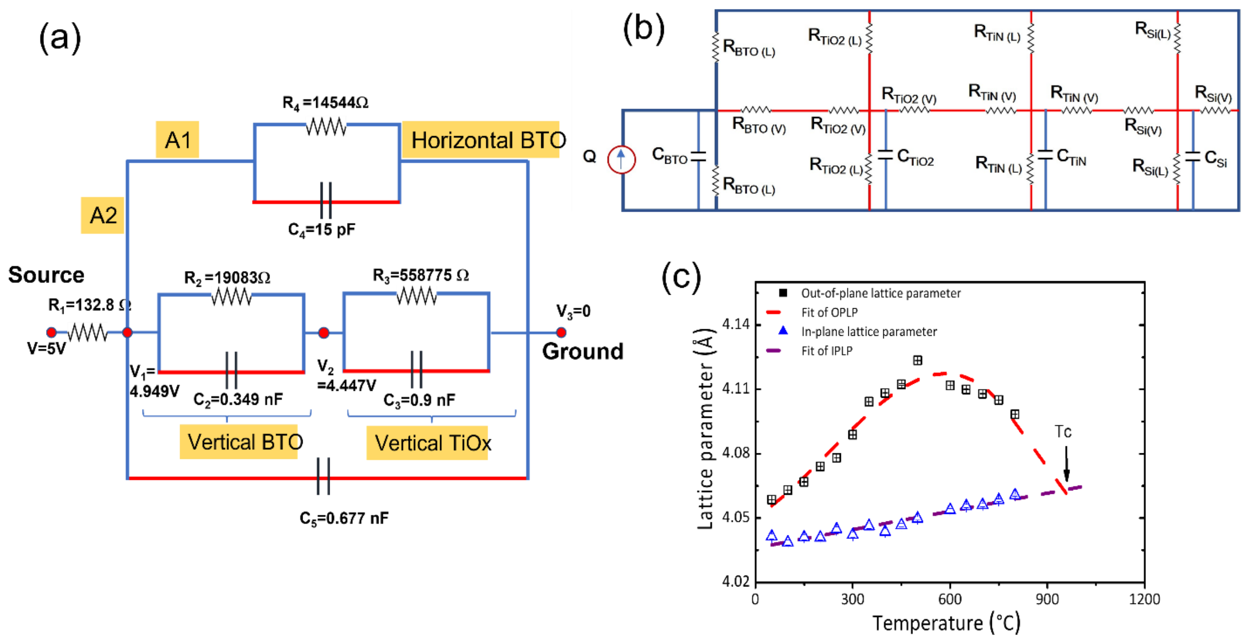

Supplementary Fig. 12: Impedance and thermal circuit models (a) Equivalent circuit used for fitting the impedance response including values of RC elements corresponding to lateral BTO layer (top path or A1) and A2 (bottom path) that includes vertical drop across BTO (V_1-V_2), TiO_x (V_2-V_3) and the MW contribution from interface obtained from Z-fit. The RC time constant of lateral BTO path is 6-7 kHz and correlates with the peak in both dielectric and electromechanical tan δ. (b) Equivalent thermal circuit model used for electrothermal simulations performed on LTspice. (L) and (V) represents lateral and vertical element respectively in each layer (c) In-situ XRD of the defective BTO/TiN/Si stack carried out to determine the coefficient of thermal expansion (Reprinted (adapted) with permission from Vura, S. *et al.* Epitaxial $BaTiO_3$ on Si(100) with In-Plane and Out-of-Plane Polarization Using a Single TiN Transition Layer. *ACS Appl Electron Mater* **3**, 687–695 (2021). Copyright 2021 American Chemical Society [5])

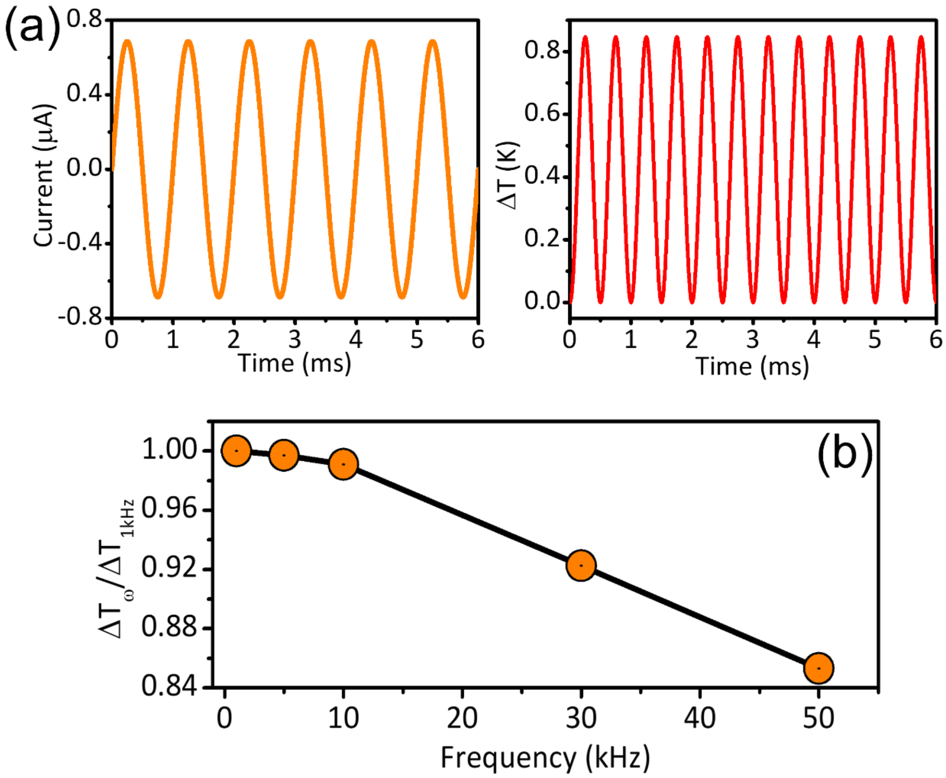

Supplementary Fig. 13: **Results of device thermal simulations on less-leaky devices:** Current waveform (from *I*-*V* measurement) used for simulating the temperature change in “less leaky” device as a function of time shown for (a) 1 kHz 5V (b) normalized temperature change as a function of frequency (ω). Maximum temperature rise is 0.8 K suggesting that Joule heating is not important in these less leaky devices for explaining the large electromechanical effect.

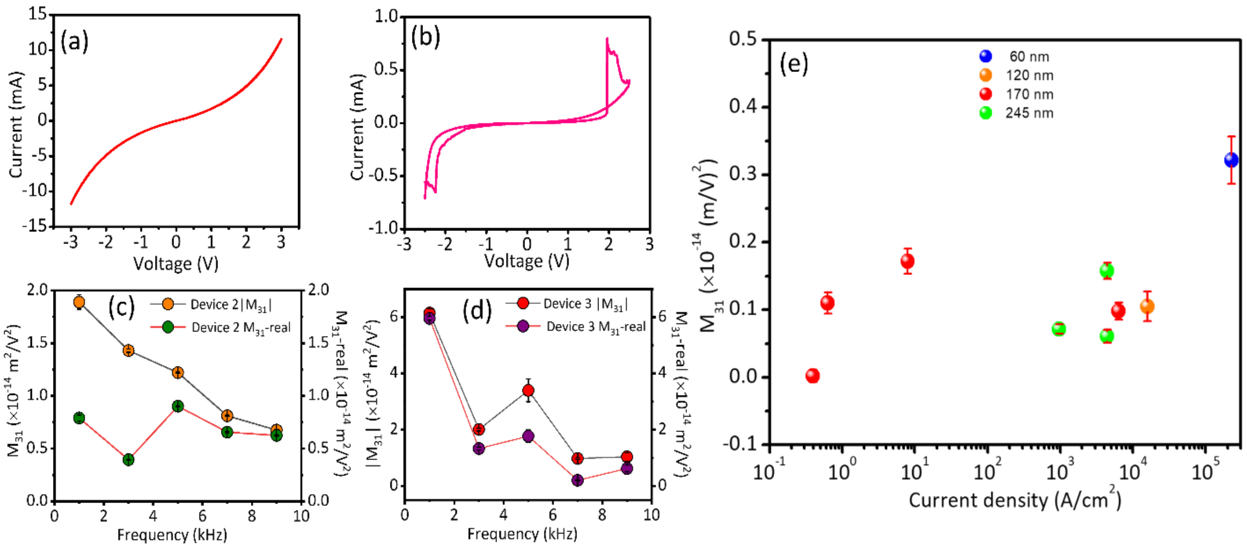

Supplementary Fig. 14: DC I-V characteristics of leaky devices and their electromechanical response *I-V* characteristics (a) and (b) from "leakier" devices showing even larger M_{31} coefficients (c) and (d) respectively which are two and six times than the M_{31} coefficients for the "less leaky" device at 1 kHz frequency, (e) M_{31} as a function of current density of measured on various thicknesses at V_{max} = 5 V and frequency of 5 kHz. Error bars in Fig (c-e) represent ± SD

Supplementary Note 13: We also did experiments on a larger set of samples with different thicknesses. However, it is not straight forward to directly compare between devices across thicknesses, without considering how leaky these devices are. In Supplementary Fig. 14e, we compile all the data, in the form of current density (leakiness) vs M_{31} measured at 5 kHz and 3 V, for IDEs on films of various thicknesses (60 nm, 120 nm, 170 nm, 240 nm). Given these large statistics, we do not see any clear trends of M_{31} with leakiness. We can say at this stage is M_{31} for various samples at 5 kHz and 3V is in the order of ~10^{-15} m^2/V^2. This provides another clue that Joule heating does not play a significant role in the observed second order electrostrain.

Furthermore, our electrothermal simulations (Supplementary Fig 15) also support that most of the displacement arises from electrostriction. Our simulations show that, at these conditions the device temperature amplitude is at the maximum 10 K (Supplementary Fig 15a), which contributes to only about 60 pm of the displacement out of 500 pm measured.

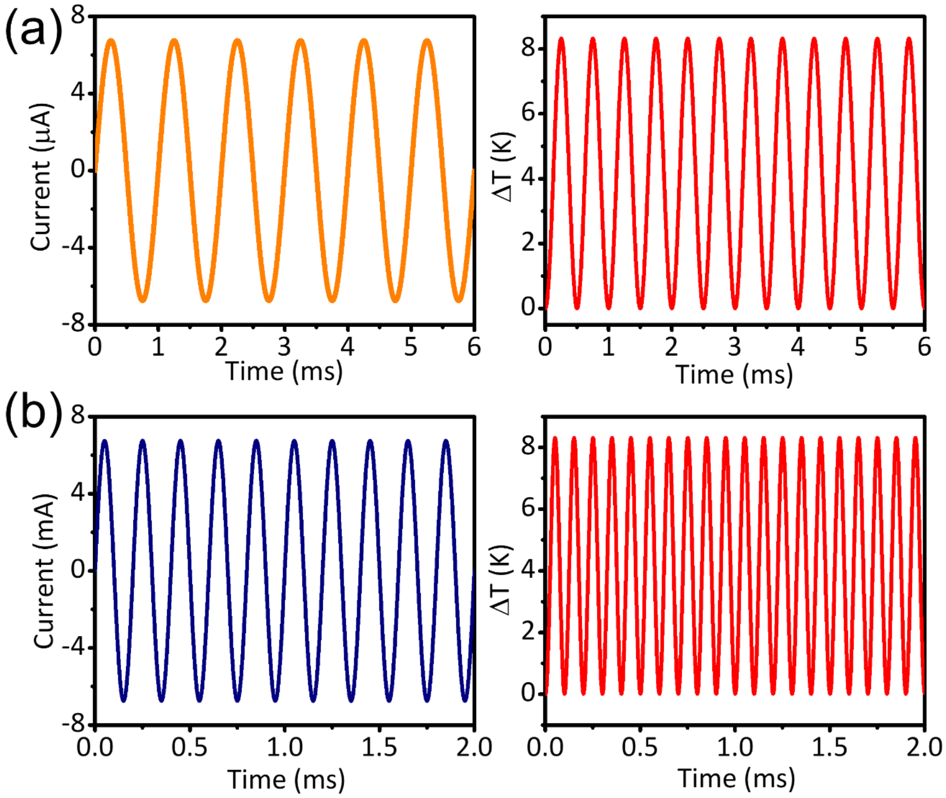

Supplementary Fig. 15: Thermal simulations on leaky devices: Current waveform (from *I-V* measurement) used for simulating the temperature rise in "leakier" device as a function of time shown for (a) 1kHz, 5V (b) 5kHz, 5V. Here we observe a maximum temperature rise of 8 K, which corresponds to a film displacement amplitude of ~60 pm. Note that the total displacement amplitude measured in this device at 1 kHz, 5 V is ~500 pm. Joule heating is still a minor part in explaining the large electromechanical effects.

Supplementary Note 14: Temperature rise in substrate and contribution to overall displacement

Ours is a dual beam measurement, with reference beam shone about a few micrometers away from the device. Thermal expansion of the substrate will not be restricted to only to the device area since heat transport occurs in longer range, and so a reference point close to the device should capture the substrate expansion and contraction with thermal cycling. However, our differential measurement should take care of it, and eliminate any substrate contribution.

Having said that, in Supplementary Fig 16a-e, we show data on five different devices of variable leakage measured at different frequencies where we show displacement measured from reference

laser, the measurement laser, and the difference signal (not differential but difference). We clearly see that the contribution from the reference laser (to be understood as substrate contribution) is negligible in all the cases.

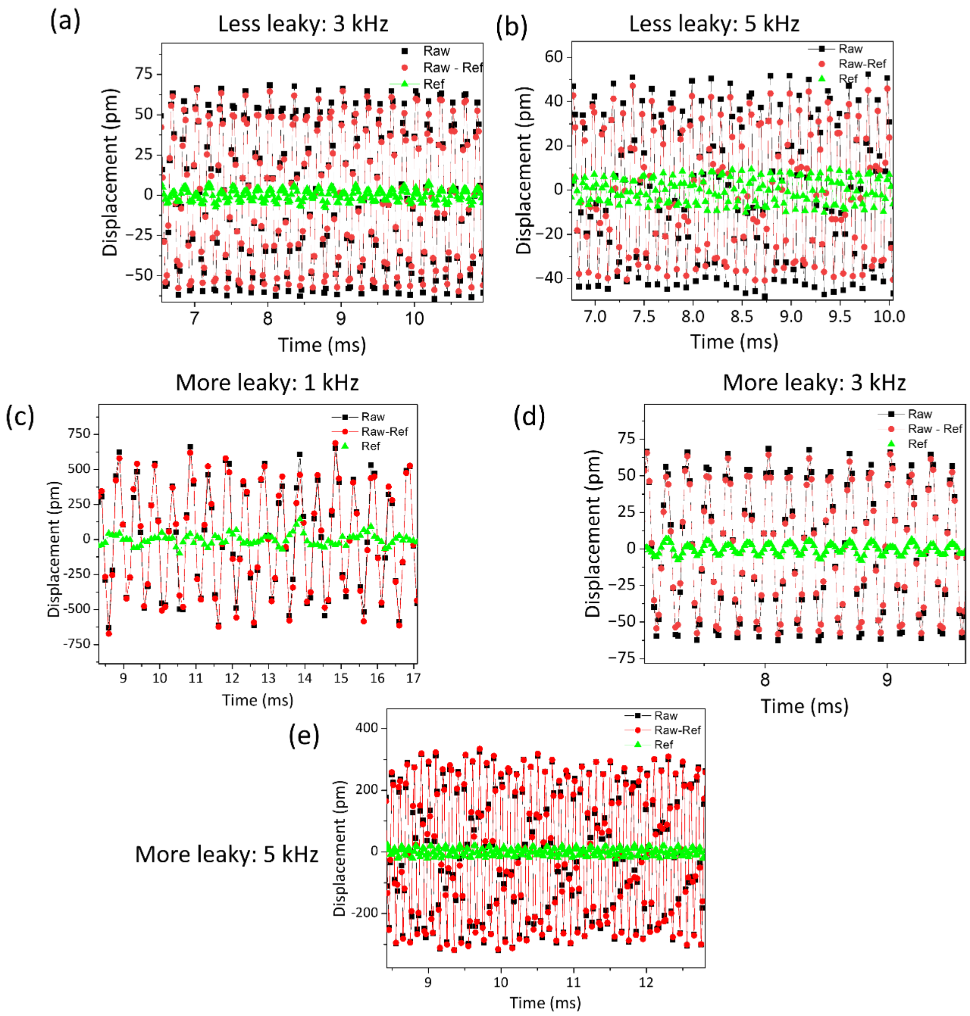

Supplementary Fig. 16: Displacement contribution from substrate: Raw displacements and reference measurement for excluding the thermal expansion effects shown for the "less-leaky" device at 3kHz (in (a)), 5kHz (in (b)), and "more-leaky" device at 1kHz, 3kHz and 5kHz (in c-e) respectively.

To exactly understand the temperature changes on the region of the substrate directly under the device, we also performed electrothermal simulations. In these simulations in Supplementary Fig 17, to model the heat transport in the vertical direction of Si, we consider Si as

(i) Only a single thermal resistor of thickness 500 µm in one case (model shown in Supplementary Fig 17a and results shown in c(left) and d(left), this does not account for various depths of the substrate being at different temperatures) and

(ii) split it as two thermal resistors in series of thickness 250 µm each (model shown in Supplementary Fig 17b and results shown in c(right) and d(right), this strategy is standard in SPICE modelling; more the sections, more precisely can the temperature at various depths of Si be modelled).

We can see that in our less-leaky representative devices, change in temperature of Si is of the order of a mK, corresponding to a displacement of 2.5 pm in amplitude at 1 kHz, and in case of leakier devices it is <10 mK, corresponding to a displacement of <20 pm at 1 kHz. The substrate temperature rise (at different points in the substrate), will be even smaller is more sections are included in the simulations. This is noise compared to the measured amplitudes of the device. In general, it is reasonable and well known that Si (substrate) is a very good heat reservoir.

These reasons show that substrate thermal expansion plays a negligible role in the effects that we have shown.

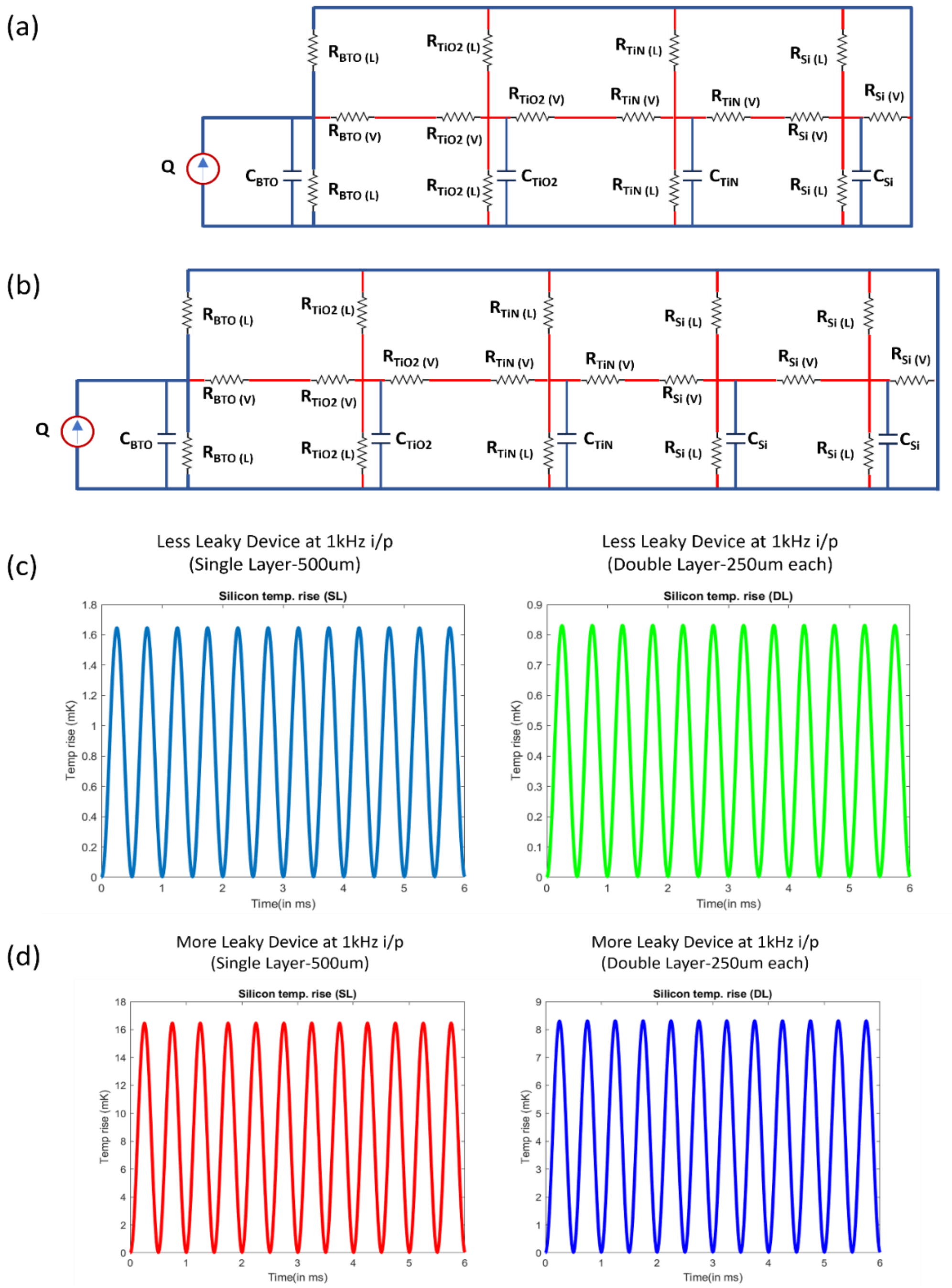

Supplementary Fig. 17: **Temperature rise in Si substrate:** Electrothermal simulations for estimating the temperature rise in Silicon substrate for (a) electrothermal circuit with single 500 µm Si layer (b) with double Si layer 250 µm each (c) simulated temperature rise for less leaky device in single (left) and double Si layer (right) (d) temperature rise for leakier device in single (left) and double Si layer (right).

Supplementary References

1. Miot, C., Husson, E., Proust, C., Erre, R. & Coutures, J. P. *Residual Carbon Evolution in BaTiO 3 Ceramics Studied by XPS after Ion Etching*.

2. Sivaramakrishnan, S. *et al.* Electrode size dependence of piezoelectric response of lead zirconate titanate thin films measured by double beam laser interferometry. *Appl Phys Lett* **103**, (2013).

3. Maruyama, K., Kawakami, Y. & Narita, F. Young's modulus and ferroelectric property of BaTiO3 films formed by aerosol deposition in consideration of residual stress and film thickness. *Jpn J Appl Phys* **61**, (2022).

4. Yu, J. & Janolin, P.-E. Defining 'Giant' Electrostriction. *J Appl Phys* **131**, (2021).

5. Vura, S. *et al.* Epitaxial $BaTiO_3$ on Si(100) with In-Plane and Out-of-Plane Polarization Using a Single TiN Transition Layer. *ACS Appl Electron Mater* **3**, 687–695 (2021).
