# Supplementary material for: Giant electrostriction-like response from defective non-ferroelectric epitaxial BaTiO3 integrated on Si (100)
Source: Nat Commun. 2024 Feb 16;15:1428. doi: 10.1038/s41467-024-45903-x (PMC10873356; doi:10.1038/s41467-024-45903-x)
Supplement: Supplementary file 3 — Description of Additional Supplementary Files [file 41467_2024_45903_MOESM3_ESM.pdf]

DESCRIPTION OF ADDITIONAL SUPPLEMENTARY FILES DOCUMENT

Supplementary Movie 1:Pedagogical demonstration of how losses create butterfly-like hysteresis in electrostriction.
